# Using ezRAD to reconstruct the complete mitochondrial genome of *Porites fontanesii* (Cnidaria: Scleractinia)

**DOI:** 10.1080/23802359.2018.1437805

**Published:** 2018-02-09

**Authors:** Tullia I. Terraneo, Roberto Arrigoni, Francesca Benzoni, Zac H. Forsman, Michael L. Berumen

**Affiliations:** a Red Sea Research Center, Division of Biological and Environmental Science and Engineering, King Abdullah University of Science and Technology, Thuwal, Saudi Arabia;; b ARC Centre of Excellence for Coral Reef Studies, James Cook University, Townsville, QLD, Australia;; c Department of Biotechnologies and Bioscience, University of Milano-Bicocca, Milan, Italy;; d Hawaii Institute of Marine Biology, Kaneohe, HI, USA

**Keywords:** Mitochondrial genome, Scleractiania, RAD sequencing

## Abstract

Corals in the genus *Porites* are among the major framework builders of reef structures worldwide, yet the genus has been challenging to study due to a lack of informative molecular markers. Here, we used ezRAD sequencing to reconstruct the complete mitochondrial genome of *Porites fontanesii* (GenBank accession number MG754069), a widespread coral species endemic to the Red Sea and Gulf of Aden. The gene arrangement of *P. fontanesii* did not differ from other Scleractinia and consisted of 18,658 bp, organized in 13 protein-coding genes, 2 rRNA genes, and 2 tRNA genes. This mitochondrial genome contributes essential data to work towards a better understanding of evolutionary relationships within *Porites*.

*Porites fontanesii* Benzoni and Stefani, 2012 is a well-defined coral species belonging to the hard-coral family Poritiidae. Although only described recently, *P. fontanesii* is a common and widespread taxon in the Red Sea, with a distribution extending to the Gulf of Tadjoura, the Gulf of Aden and Socotra (Benzoni and Stefani 2012). The genus *Porites* is still taxonomically challenging in terms of species boundaries (Forsman et al. 2009, 2017; Hellberg et al. 2016), yet *P. fontanesii* is morphologically and molecularly distinctive, presenting unique morphological features, and forming a basal monophyletic clade within the *Porites* rDNA phylogeny (Benzoni and Stefani 2012).

The individual coral sample for this study was collected at Ras Qadamah reef, in Socotra Island, Yemen (12° 41.902 N; 53° 39.683 E), and is now deposited at King Abdullah University of Science and Technology, Saudi Arabia (specimen voucher SO114). Genomic DNA was extracted using DNeasy® Blood and Tissue Kit (Qiagen Inc., Hilden, Germany), quantified using Qubit 2.0 fluorometer (Invitrogen, Carlsbad, CA), and digested with frequent cutting enzymes Mbol and Sau3AI (New England Biolabs, Ipswich, MA), following Toonen et al. (2013). ezRAD libraries were prepared using Illumina TruSeq® Nano DNA kit following the manufacture’s protocol, and paired-end sequenced using HiSeq® 4000 platform in the Bioscience Core Lab facility at King Abdullah University of Science and Technology, Saudi Arabia. Reads were assembled to *P. lobata* reference mitogenome (NC030186) using Geneious® v.10.1.3 (Biomatters Ltd. Auckland, New Zealand), and a consensus sequence exported using 0% majority option for coverage greater than 3 X. Genes were annotated using the online platforms DOGMA (Wyman et al. 2004) and MITOS (Bernt et al. 2013), and were manually inspected. tRNA was additionally scanned with the tRNAscan-SE (Schattner et al. 2005) web server.

The complete *P. fontanesii* mitogenome consisted of 18,658 bp, with the following overall base composition: A 25.81%, T 37.54%, C 13.64% and G 23.19%, in agreement with the typical mitogenome base composition (i.e. A + T rich) of scleractinian corals (Fukami and Knowlton 2005; Arrigoni et al. 2016). The reconstructed genome included 13 protein-coding genes, 2 ribosomal RNA genes (*rnl* and *rns*) and 2 transfer RNA genes (*trnM* and *trnW*). *Nad5* and *cox1* genes were interrupted by Group I Introns. *Nad5* Group I Intron consisted of 11,135 bp, comprising 10 encoding genes, while *cox1* Group I Intron was 965 bp long.

A phylogenetic tree comprising the *P. fontanesii* mitochondrial genome and all published mitogenomes of Poritidae and its sister taxon, Dendrophylliidae, has been reconstructed using Bayesian inference as implemented in MrBayes 3.1.2 (Ronquist and Huelsenbeck 2003) for 1,000,000 generations and maximum-likelihood as implemented in PhyML 3.0 (Guindon et al. 2003) (Figure 1). The monophyly of the genus *Porites* is well supported by the mitochondrial phylogeny, and a distinctive position of *P. fontanesii* within the genus is highlighted by the reconstruction.

**Figure 1.**
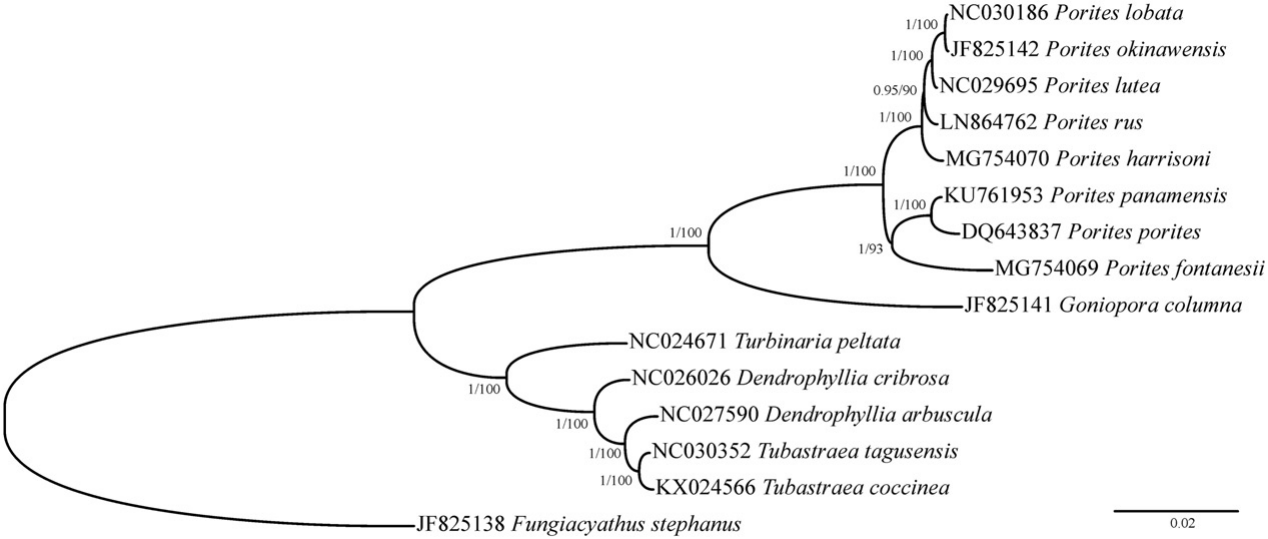
Phylogenetic reconstruction based on complete mitochondrial genomes of *Porites fontanesii* and other Scleractinia. Numbers at nodes represent Bayesian posterior probabilities and maximum likelihood bootstrap values. *Fungiacyathus stephanus* was selected as an outgroup.

The implementation of these data with other *Porites* mitochondrial genomes will help clarify evolution in one of the most important framework builders of coral reefs.
